# Remote Access to Primary Care: Theorising ‘Care Navigation’

**DOI:** 10.1111/1467-9566.70184

**Published:** 2026-04-05

**Authors:** Gemma Hughes, Sarah Rybczynska‐Bunt, Sarah O'Rourke, Sara Shasha'h, Joseph Wherton, Sara Shaw, Trisha Greenhalgh

**Affiliations:** ^1^ School of Business University of Leicester Leicester UK; ^2^ Community and Primary Care Group University of Plymouth Plymouth UK; ^3^ Nuffield Department of Primary Care Health Sciences University of Oxford Oxford UK

## Abstract

The use of digital technology to facilitate remote access to care can increase inequalities, especially for people with complex health and social care needs. Care navigation, human input to support access, is one potential solution. We aimed to understand how care navigation is practised in the context of increasing remote access in primary care in the United Kingdom. We used the concepts of social navigation and infrastructure to analyse empirical data from a study of care navigation in general practices in 2023. Our dataset included interviews with 18 care navigators. We focused on people with additional social care needs including those being homeless or insecurely housed, refugees, asylum‐seekers and vulnerable migrants, and older people requiring residential or voluntary social care and support. Our data showed the importance of people working outside of health services in a range of organisational settings. These findings allowed us to extend the concept of care navigation from a signposting and gateway role to a set of distributed sociotechnical practices. We conceptualised the problem of access as difficulty in connecting to the infrastructure of primary care. Care navigators' relational work, therefore, offered ‘nodes’ in the network of primary care: opportunities for interactions that supported connection.

## 1 Introduction

Access to general practice is high on the list of public concerns about the NHS (Allen et al. 2023) and has been a focus for UK health policy for over a decade (Simpson et al. 2015). The policy drive towards ‘digital first’, including remote access to primary care, is intended to both improve access and address general practice workload (Fit for the Future: the 10 year health plan for England 2025; The NHS Long Term Plan 2019). The increasing use of digital technologies to achieve first contact, a core component of primary care, is more convenient for some patients but can disadvantage others by creating additional barriers to primary care (Turner et al. 2022). Care navigation is a policy and practice response intended to help such patients overcome these barriers.

Sociological perspectives on the issue of access to health care, including the experiences of marginalised groups, have drawn on analysis of the negotiated social order of access (Dixon‐Woods et al. 2006) and the intrapersonal and interpersonal dynamics of health‐seeking practices (Rybczynska‐Bunt et al. 2024). We add to this body of work by mobilising two concepts, social navigation and infrastructure, to analyse qualitative data from a study of care navigation in UK general practices in 2023. We investigated the sociomaterial arrangements that allowed people with additional social care needs to access primary care remotely through digital technologies. We conceptualised the problem of access as one of difficulty in connecting to the networked infrastructure of primary care. We found that care navigators can assist marginalised people by offering ‘nodes’ in that network: opportunities for interactions that support connection to primary care infrastructures. Our study offers a novel theorisation of access to health care as being a process of navigating primary care infrastructure and, as such, offers new insights into the nature of that infrastructure.

## 2 Background

### 2.1 Primary Care Access and Inequalities

Primary care is essential to a functioning health system in achieving population health and addressing inequalities. In the UK National Health Service (NHS), general practice is free at the point of care. General practices offer primary care (including assessment, treatment, prevention, screening and vaccination) and act as a gateway to secondary and specialist health care. Access is constructed of the availability and supply of health care and the extent to which people have opportunities to gain access. Barriers to access can be shaped by financial, organisational, social and cultural factors (Gulliford et al. 2002).

Barriers to accessing primary care in the UK context have been evidenced for marginalised groups, including homeless people and refugees and asylum‐seekers (Kang et al. 2019). The unequal nature of ease of access to health care and the specific difficulties that vulnerable groups face has been conceptualised as a process of candidacy: the ways in which patients and service providers determine and negotiate eligibility for health care (Dixon‐Woods et al. 2006). Difficulties in gaining and facilitating access have been evidenced from both patient and healthcare provider perspectives (Jego et al. 2016). These difficulties can be explained, in part, by the concept of human ‘fit’ (or lack of fit) between patients and health systems (Checkland et al. 2023). Improving the fit of services means considering the appropriateness of what is offered in relation to patients' needs as well as the capacity of services. Rigid rules to manage demand can inadvertently worsen this fit (Voorhees et al. 2024); improving access requires organisational and interpersonal changes (Rybczynska‐Bunt et al. 2024).

### 2.2 Digital Access

The increasing use of digital technology to access and provide primary care remotely (e.g., through online booking, e‐consultations and remote teleconsulting and video consulting) complicates access as patients need to employ digital skills and literacy to assert their eligibility (Dakin et al. 2024). Remote access creates additional barriers for patients who are ‘digitally excluded’ due to a lack of skills, devices or connectivity (Alami et al. 2022), and it changes how patients and clinicians interact, see and know each other, for example, via the medium of video consultations (Moore et al. 2024). The loss of the shared space of the consulting room and the associated problems of the timing of remote appointments imposes greater work on patients to create the physical conditions necessary for a consultation (Humphrey et al. 2025). People who have less control over their environment due to pressures of work or insecure housing are at additional risk of being unable to access primary care remotely, with the potential for health inequalities to increase at the intersection of multiple disadvantages (Husain and Greenhalgh 2025). People with additional social needs are more likely to be digitally excluded but are also often supported to access primary care (digitally and nondigitally) by voluntary sector organisations, informal care arrangements and health services.

### 2.3 Addressing Problems of Access

Health service and policy responses that seek to remove barriers to access include organisational changes and individualised support. Organisational changes include specialist services, such as *outreach* for homeless people, to remove organisational and administrative barriers (e.g., inflexible appointment times and the need to supply proof of address to register with a general practice) and attend to social and relational difficulties such as stigma and exclusion (Gunner et al. 2019; Kopanitsa et al. 2022). ‘Mainstream’ primary care services are expected to identify vulnerable patients as part of their routine processes and, in some cases, offer navigation in the form of information and advice on services, rights and entitlements as well as relational work to build trust and engagement (MacKichan et al. 2017). Other services (typically third sector) support people to *reach* *in* to primary care by, for example, providing refugees, asylum‐seekers and vulnerable migrants with information and advocacy (Scott et al. 2022). A framework for primary care services for refugees and asylum‐seekers divides the support needed into gateway (facilitating entry), core (ensuring full registration and appropriate assessments and support) and ancillary (i.e., specific needs such as interpreting services) (Feldman 2006). Despite these efforts, barriers to access for marginalised groups persist and are exacerbated by the increasing use of digital technologies for remote access.

### 2.4 Care Navigation

Individual support for all patients along their journey into and through health services has received longstanding policy attention in the UK context over a range of initiatives, including care coordination, case management, social prescribing and care navigation. Care navigation involves supporting, guiding, signposting and referring patients to health services and other resources intended to improve their health and well‐being (Tierney et al. 2019). It is performed by a range of different people in different roles and settings. The idea can be traced to patient navigation in the United States: support given, by laypeople or healthcare providers, to people unable to access timely diagnosis and treatment due to being poor, uninsured or underinsured. This early navigation work sought to remove specific barriers to cancer care for poor people to tackle health disparities (Freeman and Rodriguez 2011). Care navigation is also needed to support patients who, due to multiple long‐term conditions, require access to a complex configuration of services, including people who need social or welfare support in addition to health care.

A broader set of practices has developed as different terms and activities have become associated with navigational work, for example, care coordinator, care navigator, community health worker, link worker, health trainer and, more recently, social prescriber (Tierney et al. 2019). In the context of primary care, care navigation involves supporting patients into, within and from primary care. Within primary care, staff match patients to the appropriate clinical contact or alternative form of support (Siddiqui et al. 2017), support patients with online consultation and triage systems to manage their own appointment processes (Chappell et al. 2023) and facilitate e‐health care coordination solutions (Kiely et al. 2021). Navigation *from* primary care involves referring patients to link workers or social prescribers who carry out relationship‐building work, support behaviour change and provide information about and referrals to relevant community services and resources (Kiely et al. 2021; Wildman et al. 2019). Care navigation, therefore, covers a range of activities in different settings aimed at supporting patients to access specific, formal health services and more general and informal resources intended to improve health and well‐being.

Our study focused on support given to patients to access general practice and therefore to make first contact with the health system in the United Kingdom. Little is known about how digital access to primary care affects care navigation or the sociomaterial arrangements that allow patients to make first contact with the health system. Taking the broad definition of care navigation noted above as our analytical starting point, we investigated how vulnerable people (those with additional social care needs) were supported to access general practice through digital and remote means.

## 3 Re‐Theorising Care Navigation as Social Navigation of Primary Care Infrastructures

Our methodological approach is informed by the concept of generativity; that is, complex phenomena, such as access to primary care, arise from the interaction of other elements, in this case people and technology (Bygstad 2017). Informed by this approach, we compare the term ‘care navigation’, which denotes a range of practices bounded by a concern with accessing services to support health and well‐being, to the theoretical concept of *social* navigation. Social navigation explains how people act in uncertain times by focusing on the interactivity between what people do and how they make sense of their social environment and the changing and fluid nature of that environment. Vigh (2009) explicates these dynamics with the metaphor of navigating a boat at sea. The fluid, ever‐changing nature of the sea means that the position of the boat has to be continually recalibrated, with adjustments made to the course to reach the desired destination. This metaphor encapsulates how people act to achieve their immediate, proximal social goals in order to attain more distal destinations. Summed up by Vigh as ‘motion squared’, the concept of social navigation allows us to recognise the changing nature, and movement, of the social environment and social formations (some of which may change more slowly than others) as well as the moves made by the agency and actions of individuals.

Considering care navigation as social navigation allows us to acknowledge not just what patients do, and are supported to do, to access health care but also how their actions interact with the changing organisation of health services. The addition of digital or remote access to health care is one such change that requires a new set of actions by patients and, for any patient unfamiliar with the requisite technology, new skills, resources or forms of support. Proximal goals, of engaging effectively with digital technology, have to be met to achieve the distal goal of engaging effectively with health care. The work that patients do to access health care, and the work that goes into supporting them, has to take account of the fluid nature of routes of access in what we might call the ‘seascape’ of primary care.

Social navigation is a powerful concept that we apply to our analysis of care navigation. Taking Vigh's notion of ‘motion squared’ (i.e., motion within motion) as our starting point, we retain the concept of navigation as shorthand for both the proximal and more distal practices undertaken in the pursuit of access to health care, both those undertaken by patients and by people (care navigators) supporting patients. However, we bring the metaphor of the boat on the ocean (to indicate the fluid nature of social formations through which people navigate) into dialogue with a fluid and emergent conceptualisation of infrastructure. We offer this as a more apt metaphor for the material nature of primary care ‘barriers’ to access. However, although infrastructure is sometimes depicted in static and material terms (e.g., as roads, buildings, pipes, wires and so on), sociologists view infrastructure as sociomaterial, relational and dynamic. In this sense, infrastructure is the uncertain and ever‐changing ‘ocean’ which the service user must (be supported to) navigate.

To examine this infrastructure, we draw on Star's (1999) seminal work and on more recent theorisations of chronic care (Langstrup 2013) and health information infrastructures (Greenhalgh et al. 2019). Infrastructure is not discrete from human organisation but is emergent from and contributes to how people work, live and organise themselves. A fundamentally relational concept, infrastructure is relevant to people as they perform certain practices, often invisible until it breaks down. In the context of chronic care, where people are supported to live at home over long periods of time, infrastructures are constituted of inconspicuous or mundane elements such as medication, phone calls and daily routines. Embedded in everyday life, these elements form an ongoing sociomaterial connection between patients at home and the clinic. These connections are not static; navigating them requires ongoing work, and they have ‘place‐making’ effects, for example, reordering things and people in the home (Langstrup 2013). We build on these ideas to consider how primary care infrastructures are made apparent through the actions of patients and those supporting them as they seek to access health care. Greenhalgh et al.’s (2019) revisiting of Star's characteristics of infrastructure guides us to consider the extent to which primary care infrastructures are materially scaffolded; embedded, relational and emergent; collectively learnt, known and practised; patchworked and path‐dependent; and institutionally supported and sustained.

## 4 Methodology

Substituting the metaphor of the sea for the sociomateriality of primary care *infrastructure*, we investigated the social navigation of accessing general practice. We traced the ways in which care navigators supported people to engage with the social and material features of technologies and health care services. To organise our empirical findings, we used the conceptual tool of narrative networks (Pentland and Feldman 2007). Narrative networks describe relationships between technologies and organisational forms and the interactions between people and technologies that create patterns of activities that allow organisations to function. Narrative refers to the purpose and intention of the patterns of activity; in our study, the intention of accessing primary care from different organisational settings. Networks refer to the multiple ways in which combinations of actions can occur; for example, a patient might book an appointment online, by phone or by asking someone else to make an appointment on their behalf. These actions and interactions form the nodes of the network. This approach to tracing interactions between people and technologies owes much to actor‐network theory, which, in broad terms, conceptualises the social world as being comprised not only of people (as agents or actors) but also constituted of actions and interactions between people and material objects. People and technologies are understood to be ‘actants’ of social processes; networks of actants are therefore the stuff of social life (Latour 2005). Our focus, however, follows Star and Vigh in being primarily interested in the relational activities of human actors which comprise these nodes. We mapped nodes to create narrative networks that represented the social navigation of patients using digital (and nondigital) forms of access to general practice.

## 5 Methods

The overall aim of this study was to investigate digital care navigation for people with additional social care needs. We asked the research question: What sociomaterial arrangements allow for people with additional social care needs to access primary care remotely through digital technologies? Digital technologies used to access primary care in our dataset included web‐based portals, email consultations and telephone contact.

### 5.1 Setting and Sample

The study took place in the United Kingdom during 2023. Ethical approval was granted by the East Midlands—Leicester South NHS Research Ethics Committee and UK Health Research Authority (September 2021, 21/EM/0170) and subsequent amendments. All research participants gave informed written consent. We recruited 26 research participants: 18 care navigators, 5 stakeholders (people involved in strategic/policy work on care navigation) and 3 social prescribers (a role related to care navigation increasingly supported in policy and practice). We recruited care navigators working with people known to face barriers in accessing remote health care, namely, homeless people, refugees/asylum‐seekers and older people including those residing in care homes. The 18 care navigators were not named as such in their job titles; rather, they had a range of responsibilities (see Table 1) which included care navigation work (e.g., information and advice on GP registration, booking appointments and organising consultations) alongside, or incorporated into, their other duties. They were recruited from practices involved in a larger programme of research on remote access, the study design for which has been reported elsewhere (Greenhalgh et al. 2022; Hughes et al. 2023). Ten participants were recruited from these practices and a further 8 from organisations known to those practices through ‘snowballing’, that is, suggested by interviewees as potential participants. Stakeholders were identified by their roles in policy and practice and through the expert advisory group of the wider study. Members of the focus group were recruited from 2 of the 9 practices connected to social prescribing services.

**TABLE 1:** Research participants' organisational settings and roles.

	Research participant	Organisational setting	Role
1	Care navigator interview	General practice	Practice manager
2		General practice	Reception team leader
3		General practice	Reception team member
4		General practice	Reception team member
5		General practice	Reception team member
6		General practice	Reception team member
7		General practice	GP partner
8		General practice	Patient care advisor
9		General practice	Health coach
10		General practice	Social prescriber
11		Voluntary sector organisation	Social prescriber
12		Care home	Care home manager
13		Charity (lunch club)	Activity facilitator
14		Charity (refugee service)	Volunteer GP
15		Charity (refugee service)	Volunteer
16		Homeless outreach service	GP
17		Homeless hostel	Hostel worker
18		Call centre	Team manager
19	Stakeholder interview	Health policy organisation	Digital lead
20		Social care policy organisation	Digital lead
21		National patient organisation	Policy lead
22		NHS England	Digital inclusion
23		Digital health provider	Director
24	Focus group	Primary care network	Social prescriber
25		National Academy for Social Prescribing	Social prescriber
26		Voluntary sector organisation	Social prescriber

### 5.2 Data

Qualitative data comprised 18 care navigator interviews (9 online and 9 in‐person ‘go‐along’ interviews, which included observations of service settings and daily routines), video interviews with 5 stakeholders and an online focus group with 3 social prescribers. Interviews were conducted by GH, SRB, SOR, and SS'h. The focus group was conducted by SRB, SOR, and SS'h. Audio recordings were made of interviews (with contemporaneous field notes made of go‐along interview observations) and the focus group, which were transcribed.

### 5.3 Member Checking

An online co‐production workshop with a further 19 stakeholders tested and refined findings and identified implications for practice and policy. Invitations to the co‐production workshop were shared through the study's networks. The workshop was facilitated by Authors SRB, SOR, and SS'h and contemporaneous notes were made.

### 5.4 Analysis

**FIGURE 1:**
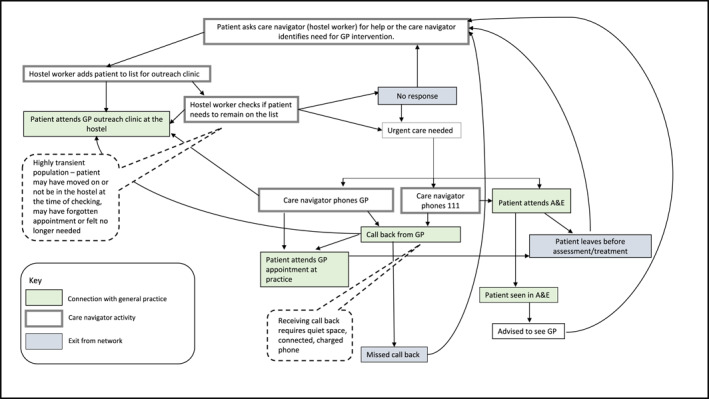
Narrative network of care navigation from a homeless hostel.

**FIGURE 2:**
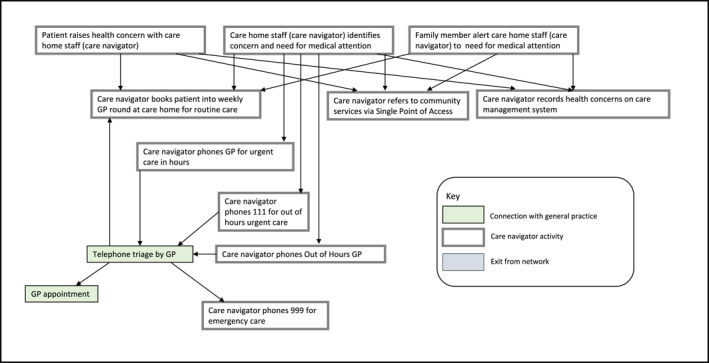
Narrative network of care navigation from care home.

**FIGURE 3:**
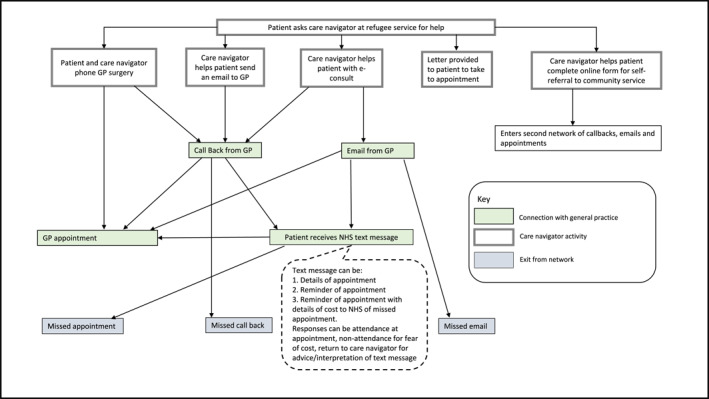
Narrative network of care navigation from refugee support services.

There were three stages of analysis. First, the authors mapped a generic care navigation pathway, asking: Who does care navigation? Where is it done, how and with what tools and resources? Having divided the pathway into pre‐appointment booking, appointment booking, appointment allocation, consultation and referral onwards, we honed in on pre‐appointment and appointment booking as where navigation into general practice occurred. Second, we summarised our data by drawing (initially using pen and paper) narrative networks in three distinct organisational settings (homeless hostel, refugee advice service and care home) to identify actants and interactions. Actants were the people (homeless hostel workers, refugee advice service volunteers and staff, care home staff and patients) and technologies (computers, phones, tablets and internet access provided by organisations, individual care navigators and patients). Mapping the sequence of events diagrammatically in different organisational settings showed how interactions between care navigators, patients and technologies enabled contact to be made with general practice, including the sequences of events needed to achieve first contact and the actions that could progress or alter those sequences (or *nodes* in the network) (see Figures 1, 2, 3). Details of how interactions might cause a lack of progress in the network were also included. Finally, the team presented the narrative networks at a co‐production workshop of 19 stakeholders, including social prescribers, general practice managers, homeless hostel workers, refugee services and link workers, to test and refine the networks.

## 6 Findings

### 6.1 Overview

First, we explain the general narrative about how access shifted from in‐person to remote and how this changed the nature of the interactions (e.g., contacting a GP receptionist) required to achieve certain functions (such as booking an appointment). We then present different narrative networks of access to general practice from the organisational settings of a homeless hostel, a care home and a refugee advice service. Finally, we conceptualise the sociomaterial practices of care navigation as offering pre‐primary care support for patients to make first contact with general practice, facilitating synchronicity and enabling connection with the ‘heavy’ technology of primary care infrastructures.

### 6.2 Changing Routes of Access: From In‐Person to Remote

The changing nature of the primary care infrastructure was characterised across multiple sites in our study by the pandemic‐induced instigation of phone‐first contact. These arrangements were typically superseded by hybrid systems intended to offer remote access (incorporating phone, email or web‐based contacts) but, in practice, providing some form of workaround by way of ‘walk‐ins’ for vulnerable groups. One interviewee explained the evolution of a system of accessing primary care for homeless people:

… So before the pandemic, we would have a system whereby we would ask people to attend either eight o'clock in the morning or one o'clock in the afternoon for on the day appointments. So half of our appointments, so it's kind of walk in like that, and half of them were pre bookable … Obviously, when the pandemic came along, we couldn't then tell people to turn up because that wasn't allowed. So we then had to start issuing people with telephones, so that they could phone us eight o'clock in the morning or one o'clock in the afternoon, to book their on the day appointments … I think that through that we were trying to sort of equalise people's access by phone … So I think we've now reached a point where we are kind of slightly hybrid, we try very hard to make sure that people don't experience digital exclusion. So we now say that people can either come in or phone up at the time. But obviously, there's still a slight disadvantage there. Because it's still always easier to phone up … if you're somebody who's got accommodation, you can sit at home in your flat. Whereas it's more difficult to fit it into your day, to time it perfectly correctly … to time your appearance at the surgery, exactly the right time, to get an appointment [when you are homeless].

(Homeless outreach GP)

The use of e‐consultations and web access was understood by some participants working within general practice as offering the potential to help manage the excessively high volume of phone calls received, as one GP partner explained:

A couple of years ago we had a thousand calls coming into the practice a day and it’s just unmanageable and that kept growing like that so we made the transition to online … [the call centre] were essentially a facilitator for someone needing to fill in a form to access us.

(GP partner)

And as a reception team member elaborated:

… every call comes into reception, ordering prescriptions, queries, blood results … sorting things for the district nurses, everything comes through to us …

(Reception team member A)

Although online access made it easier (in some respects) for general practices to manage the volume of contacts, participants were aware that remote booking of appointments could make things more difficult for some patients:

I take daily bookings from patients, interact with them every single day. The way that I would see with booking in remotely … I think it’s a really good thing but … for older patients and obviously for people that are homeless they don’t have resources to use, plus the knowledge of how to use them. But within our surgery they’ve got the chance to call in, they can come in and be seen face to face as well as eConsultation … we are offering several options for patients to make contact with us.

(Reception team member B)

Such workarounds to the ‘digital first’ approach were common across our dataset as general practices sought to identify and ‘flag’ patients who needed direct access (such as vulnerable patients, people at the end of life and their supporting family members) or proactive engagement (including patients receiving cancer diagnoses). These responses from general practice to the difficulties some patients faced were similar to the provision of outreach clinics, which bring appointments closer to patients by reducing the number of interactions and offering the option of ‘walk‐ins’ for certain patients. These in‐person, synchronous interactions require less navigational support.

From the patient perspective, changing routes of access to appointments and communication with general practice represented wider changes of increased digital technology, as we found when observing a lunch club for older people. This comprised a weekly meeting with quizzes, entertainment and, of course, lunch, run by a charity with a small number of paid staff and volunteers. Volunteers provided general support with web‐based information, showing people how to use the internet but also to circumnavigate digital access to services such as general practice or the local council by finding phone numbers to call or addresses to write to as alternatives to online portals.

Overall, the challenges that some patients experienced with digital access were addressed by interactions with care navigators that varied according to the organisational context.

### 6.3 Different Narratives of Access

The organisational settings of the homeless hostel, the care home and the refugee advice service each had different relationships with general practice, institutional structures, resources and ways of providing care navigation.

Both the homeless hostel and the care home had formalised arrangements with general practices whereby GPs would offer regular clinics or ‘rounds’. In both settings, the primary care infrastructure reached into and was supported by the respective institutions.

A weekly outreach clinic at the homeless hostel brought the otherwise distal destination of an appointment with a general practitioner into close proximity with the potential patient. This reduced the number of interactions and therefore the social (as well as the geographical) distance that the patient needed to travel. In principle, the narrative of accessing general practice in this context was straightforward: requiring only an interaction between the potential patient and a hostel worker, resulting in the patient being put on the weekly clinic list. The next interaction could be the patient's attendance at the clinic, thus achieving first contact. However, our data showed that, for many people, additional interactions were required (see Figure 1). For example, to prevent scarce appointments being allocated to people who would not attend, hostel workers would check shortly before the weekly clinic that people (who were highly transient) were still in residence. People were removed from the clinic list if they did not confirm (see detail of Figure 1). If they still needed to see the doctor, they would have to start the process again, leading to repeated interactions. Also, some people forgot, or decided they no longer needed, the appointment on the day of the clinic, even if they had previously confirmed. Further, additional nodes were added to the network if urgent care needs arose. The network was complicated, with multiple nodes and opportunities for the narrative to be extended, undergo repeated loops and fail to achieve the goal of a primary care appointment.

In contrast, the weekly GP ‘round’ in the care home was for a stable population. The narrative was straightforward; once an appointment was booked by care home staff for a resident, it would usually be successfully completed. In addition, there were other routes to health care via direct access to community services from the care home. An electronic care management system supported information exchange with the GP who would also conduct medication reviews and advise care home staff on patient care when attending the home. The network for care home residents was institutionalised and relatively simple with few nodes (see Figure 2) and therefore reduced opportunities for breakdown or failure to achieve the goal of securing health care.

The narrative of access for refugees and vulnerable migrants to general practice was more emergent, comprising referrals, advice and advocacy. We studied care navigation work undertaken by volunteer doctors at a refugee health service. The service was run by a small charity and offered drop‐in sessions for people seeking health care. In this context, volunteer doctors could use their medical knowledge to offer information but not treatment or formal access to health care (e.g., they could not prescribe medication or order diagnostic tests). Instead, the doctors would identify urgent medical needs and advise how to access general practice or other services which accepted self‐referrals (mental health counselling and physiotherapy were two such services available in this location). Interpersonal skills were necessary to build trust with people seeking help, but here care navigators also brought their medical and health service knowledge to the interactions in the network. On some occasions, the volunteer doctor would attempt to strengthen the narrative network in the form of a letter conveying their medical assessment for the patient to take away with them if, for example, they were concerned that the patient might not be able to communicate effectively with another practitioner (see Figure 3). Navigating this form of emergent, ‘patchworked’ primary care infrastructure was contingent on care navigators sharing their knowledge and rested on in‐person rather than remote support.

The organisational settings of homeless hostels, care homes and refugee advice services provided different contexts for the narrative of accessing primary care, each forming a different infrastructure through which a patient navigated. Next, we compare the common actions that patients took to achieve first contact with primary care, manage callbacks and connect to technology.

### 6.4 Care Navigation ‘Pre‐Primary Care’: Support to Make First Contact

We found that primary care was distributed beyond the confines of general practice through formal and informal outreach activities. Additional care navigation work was required for people who were not registered patients. These people were socially marginalised (e.g., they were residing in homeless hostels or were vulnerable migrants) and needed care navigation that was ‘pre‐primary care’, a critical precursor to achieving first contact with general practice.

Despite the physical presence of a weekly GP clinic in the hostel, achieving first contact could require repeated interactions between homeless people and hostel staff, with multiple nodes offering opportunities for the network to expand and thus increase the distance to the goal of securing health care. The number of nodes in this network (Figure 1) shows how the necessarily flexible modes of engagement in health care also provided opportunities for people to change their mind, forget, move on or otherwise disengage from the sequence of actions needed to make first contact with general practice. The relational nature of the infrastructure was apparent when considering how the quality of the interactions represented by the nodes in the network facilitated consistency and continuity through the building of rapport and trust. Outreach from general practice into the refugee advice services was more informal, without institutional support. As a ‘drop‐in’ service, there was less time available, and fewer opportunities, for patients to meet with care navigators. This more emergent arrangement involved the distribution of medical knowledge beyond general practice through advice, information, letters of support and referrals, as volunteers drew on their clinical expertise and knowledge of health to advise, inform and support patients. Care navigation in both the homeless hostel and the refugee advice service was characterised by flexible arrangements, with care navigators demonstrating supportive, consistent and rapport‐building interpersonal skills and significant knowledge of primary care and the wider health care and welfare system.

Care home residents, in contrast to the marginalised patients seeking first contact with general practice, were already registered patients. For these people, care navigation revolved around support in using technology and understanding the changing processes of gaining remote access to general practice, for example, in booking an appointment (see Figure 2). The infrastructure of primary care was well supported and integrated with the organisational setting of the care home.

### 6.5 Care Navigation as Synchronicity: Managing Callbacks

A common feature of remote access to general practice was the ‘callback’: a phone call, email or text message response from general practice to a patient's request for an appointment. The callback is an intrinsic component of remote access to general practice and presented significant problems for some people represented in our dataset due to the interconnected problems of location and timing: a lack of synchronicity.

For example, vulnerable migrants seeking advice at the refugee health service would be supported to make first contact with a general practice but would not usually be able to wait at that location for a callback. The advice service was busy, with many people needing to use the facilities, and had limited rooms available for confidential discussions. This meant that patients would usually receive a callback on their own device once they had left the service and had no immediate access to help with translation or comprehension of the call. A text message, as used by some practices, comprising an appointment slot could be more easily translated from English than a phone call. If the patient received the callback and attended the appointment, first contact would be achieved (see Figure 3). However, the process of access was complicated if the callback was missed. This narrative network presented multiple opportunities for patients to miss appointments due to a lack of synchronicity between patients' ability to make contact (from a stable location with appropriate support) and the practice's ability to respond (at a different point in time). A further communication problem arose when GPs included a warning about the cost to the NHS of missed appointments in their callback or reminder text. With no one readily available to interpret this text, some patients misunderstood this message, believing it was telling them the price of their appointment, which led to them not attending for fear of the cost.

The problem of lack of synchronicity arose for the homeless people in our dataset when they required care more urgently than through the weekly outreach clinic provided in the hostel. On these occasions, patients might attend Accident & Emergency (A&E), or a hostel worker might call NHS 111 (a free phone or online urgent healthcare advice service). Some patients would then need to wait for a callback from 111. For a homeless person to respond to a callback, they would need to remain in the reception area of the hostel for an unspecified period of timeand to receive the call would need an appropriate quiet space at the precise moment of the call, as well as having access to a (fully charged, Wi‐Fi‐enabled or mobile‐networked) phone. The introduction of the ‘callback’ process from general practice to a homeless person without access to their own device or a fixed location added additional nodes to the network (see Figure 1) and further opportunities for them to ‘exit’ the network and not reach their desired destination of an appointment with a primary care practitioner.

In contrast, people residing in a care home were in a stable location for both weekly clinics and callbacks, and there were staff consistently available to take calls on their behalf, demonstrating institutional support for the primary care infrastructure. The stability of this network was found in the consistency of the relationships between staff and patients, and the stable material arrangements of time and space that allowed for the necessary interactions between staff, patients and technologies to produce access to primary care.

### 6.6 Care Navigation and Technology: Relating to the Primary Care Infrastructure

Having considered how narrative networks were constituted by interactions in sequence, we now turn our focus to how people engaged with technology during those interactions and how this engagement allowed for navigation across the primary care infrastructure. We found that interactions between care navigators and patients included both ‘lightweight’ and ‘heavyweight’ technology. Lightweight technology, such as language translation apps on smartphones, facilitated care navigator–patient interactions. Patients needed navigational support to use the heavyweight technology, such as online self‐referrals, required to access services. In the refugee health service, for example, care navigators would not always share a common language with the people they were advising. Interactions would often be mediated by translation software with both parties using their own devices. This use of technology offers an example of what Bygstad (2017) calls lightweight technology; it is light in terms of being mobile, cheap and easy to use but, more importantly, it does not require any knowledge of, or connection with, institutional information and communication systems. Rather, it is driven by the user's needs for solutions enabled by ‘consumerisation and innovation’.

In comparison, ‘heavyweight technology’, such as an electronic self‐referral form, requires understanding the ‘knowledge regime’ or work practices and conventions associated with organisations. Some services in our dataset, including general practices, would accept self‐referral from patients via a web‐based form which asked for details of the individual, their presenting complaint and an email address to which the response from the service would be sent. This form, and the associated communications, revealed aspects of the infrastructure of primary care which were difficult for marginalised people to penetrate. The questions on the form used language that required literal translation (e.g., by a language app) and an understanding of the classification of symptoms that the receiving organisation used to assess eligibility for their services. Here, the care navigators could assist by translating the concerns of the patient in front of them into language that fitted into the form and the service criteria. Care navigation in this context involved sharing the knowledge regimes of the UK health system.

Care navigation also required facilitating a sociomaterial connection with the infrastructure of primary care. Patients (in our dataset, this included those attending the refugee service) had to provide an email address to access certain services. Unlike the ‘noninvasive’, lightweight technology of a language app that facilitated a particular interaction, the use of an email required greater ongoing communication with and integration into the health systems’ organisational information and communication architecture. Additional resources were required, comprising the knowledge of how to create an email account, the requisite personal details including an address and a phone number to verify the account, and the material resources of devices, connectivity, power and data. In contrast, care home residents were already connected to such an organisational ICT architecture; care homes had their own form of electronic records which, although not necessarily interoperable with general practice, provided consistent access to information about each resident and their health care. In the care home setting, access to primary care was incorporated into the support people received by virtue of their residency in that institution.

In sum, comparing the different organisational settings found in our data allowed us to identify aspects of primary care infrastructures and the work that care navigators accomplished to support patients to connect with those infrastructures. Primary care was distributed materially, located in outreach clinics and ward rounds, and the relational and knowledge‐based nature of the infrastructure was evident through networks of support. We found that additional ‘pre‐primary care’ navigation work was required to support people (including homeless people and vulnerable migrants) not registered with a general practice to make first contact. The difficulties faced by some patients in receiving a ‘callback’ from general practice were experienced to a greater extent by homeless people and vulnerable migrants than by those in more stable social situations and required care navigation support to bridge gaps in the material and relational infrastructure of primary care.

The introduction of remote access to primary care was experienced by people with social care needs not as a more streamlined and efficient replacement for previous modes of access, as might have been intended, but as an additional set of interactions to navigate. The location of these interactions when ‘remote’, that is, no longer located in a specific general practice, brought a range of challenges for people who did not have access to consistent, uninterrupted support in a static place. Here, care navigation involved support with multiple proximal interactions (such as online registration, e‐referral forms and telephone appointments) that constituted nodes in networks of access to the distal goal of primary care. In contrast, a more stable network was apparent when care navigation was incorporated into institutional support, such as that provided by care homes for older people, where proximal interactions were readily and consistently achieved within a fixed time and space.

## 7 Discussion

We used the concept of narrative networks to analyse how access to primary care was generated by the interactions between people and technologies. Our analysis offers new insights into both care navigation and the nature of the primary care infrastructure across which people navigated to access health care. Our study offers a novel theorisation of access to health care as being a process of navigating primary care infrastructure and, as such, offers new insights into the nature of that infrastructure. We found primary care infrastructure to be unevenly distributed across networks rather than being located within the confines of general practice, raising questions about the extent to which primary care can be said to offer ‘first’ contact as well as the extent to which remote access contributes to the infrastructure.

Although previous studies have analysed the negotiated social order of accessing care (Dixon‐Woods et al. 2006), intrapersonal motivations (Rybczynska‐Bunt et al. 2024) and the ‘fit’ between people and services (Checkland et al. 2023), our use of narrative networks allowed us to map where and when those negotiations and interactions took place, and in what sequences, to allow patients to move towards their goals. Previous studies of digital and remote access to primary care have highlighted the dangers of increasing inequalities, including through the difficulties created by the ‘displacement’ inherent to remote care (Humphrey et al. 2025). Our empirical findings show the additional work required beyond the ‘gateway’ to health services. Our conceptual work shows how care navigators can create nodes in a network of interactions, supporting the social and geographical movement of patients across that network. The ability of care navigators to create these nodes was contingent on being able to offer a stable point of contact, which allowed for repeated interactions if necessary (such as those observed in the homeless hostel) and that involved shared time and space to avoid the problem of the callback. In‐person contact was necessary, if not sufficient, to create these nodes which allowed patients to connect to the infrastructure of primary care. Interpersonal skills and relationships of trust were also needed, with ongoing work required on the part of care navigators to sustain the nodes in networks and hence for patients to access care.

We add to knowledge of ‘barriers’ to access by showing how interactions that constituted nodes in the network of access involved the sharing of knowledge resources. We found these were not simply resources about how to use digital technology; rather, they were sociomaterial resources necessary to navigate the infrastructure of primary care: for example, knowledge of eligibility criteria for services embedded in the use of online referral systems, the language of diagnoses and symptoms included in a letter to take to a future appointment, and the creation of an email address that allowed connectivity with the information architecture.

Material, relational, knowledge and institutional characteristics of the primary care infrastructure contribute towards creating or addressing difficulties in access for people with additional social care needs. The need for these resources to navigate remote access was not evenly spread across our dataset, indicating that the primary care infrastructure was unevenly distributed to different groups of people and in different organisational settings. Institutionally supported primary care infrastructure was easily accessed by people residing in care homes, with care navigation being provided across simple, short networks within a stable physical environment. Less material scaffolding for the primary care infrastructure was available to people in more insecure situations (including homeless people and vulnerable migrants). There were fewer opportunities for care navigators to have in‐person interactions with people seeking health care due to the limited availability of physical space. Networks to access health care therefore became longer, more complicated and fluid, requiring more work on the part of care navigators to engage people in relating to, and knowing about, the infrastructure. For people marginalised by their lack of connection to the primary care infrastructure, ‘first contact’ was not their appointment with a GP but their interactions with care navigators who facilitated access to general practice.

## 8 Strengths and Limitations

Narrative networks allowed us to analyse the detail of specific interactions. The value of these networks as an analytical device is their flexibility to represent both what did happen and what could happen at different junctures. However, the networks produced are an interpretation of events that included only those interactions we were able to observe or reconstruct from accounts provided in interviews. They are inevitably both a ‘tidied‐up’ representation of the complexity of social life and one that serves our exogenous focus (i.e., as part of the narrative of achieving access to health care) rather than one that represents the perspective of the patients involved, for whom those interactions would potentially have different meanings.

There are three key findings from this study that have particular implications for policy and practice: Providing digital access to primary care complicates the network of access, increasing the number of potential interactions required; supporting people to navigate digital access requires synchronous (primarily in‐person) support; and significant work is required beyond health services to support access to primary care. ‘Digital first’ policies without sufficient care navigation support carry significant risk of marginalising people with additional social care needs.

## 9 Conclusion

Remote access through digital technology has become patchworked into the primary care infrastructure that we studied in the United Kingdom. Some people are served well by these additional routes to general practice, as they have the sociomaterial resources and/or the institutional support to connect with the infrastructure. Others are not served so well by virtue of their more tenuous sociomaterial circumstances. Here, care navigation can assist with the achievement of their goal to access health care through informed, skilful interpersonal, in‐person interactions.

## Author Contributions

**Gemma Hughes:** conceptualization, formal analysis, funding acquisition, investigation, methodology, writing – original draft, supervision. **Sarah Rybczynska‐Bunt:** conceptualization, investigation, funding acquisition, writing – review and editing, methodology, formal analysis, supervision. **Sarah O'Rourke**: data curation, formal analysis, investigation, writing – review and editing. **Sara Shasha’h:** data curation, formal analysis, investigation, writing – review and editing. **Joseph Wherton:** methodology, writing – review and editing. **Sara Shaw:** funding acquisition, writing – review and editing. **Trisha Greenhalgh:** funding acquisition, supervision, writing – review and editing.

## Conflicts of Interest

The authors declare no conflicts of interest.

## Data Availability

The data that support the findings of this study are available upon request from the corresponding author. The data are not publicly available due to privacy or ethical restrictions.
